# Windows for intervention in the prearthritis phase of rheumatoid arthritis? A narrative review of key triggering events and potential preventive strategies

**DOI:** 10.1136/rmdopen-2025-005778

**Published:** 2025-07-10

**Authors:** Alf Kastbom, Sara Turcinov, Vivianne Malmström

**Affiliations:** 1 Division of Inflammation and Infection, Department of Biomedical and Clinical Sciences, Linköping University, Linköping, Sweden; 2 Division of Rheumatology, Department of Medicine, Center for Molecular Medicine, Karolinska Institutet, Solna, Sweden; 3 Theme of Inflammation and Ageing, Medical Unit Gastro, Derma, Rheuma, Karolinska University Hospital, Stockholm, Sweden

**Keywords:** Arthritis, Rheumatoid, Autoantibodies, B-Lymphocytes, T-Lymphocytes

## Abstract

Rheumatoid arthritis (RA) is an autoimmune disease that often evolves over several years, which in the presence of risk factors, is termed the at-risk phase. Several of the currently approved treatments for RA have been evaluated for the prevention of RA during the at-risk phase, but without showing effects sufficient to warrant their use prior to diagnosis. There is an ongoing surge in research efforts to understand mechanisms underlying the onset of RA, in particular concerning deviations in the adaptive immune system and the role of mucosal surfaces in the breach of self-tolerance and triggering of arthritis. With this focus, we here aimed to review current knowledge on RA development prior to arthritis onset. Also, since the pre-arthritis phase may contain windows of opportunity to prevent RA onset, we present and conceptualise different strategies to potentially interfere with steps leading to RA. While the body of knowledge is increasing, our understanding of RA development remains incomplete, and available studies highlight that RA appears similarly heterogeneous prior to onset as is seen after diagnosis. Hence, the timing and the selection of subjects will be crucial for the success of future potential interventions.

**WHAT IS ALREADY KNOWN ON THIS TOPIC**

- Rheumatoid arthritis (RA) often develops over several years, where genetic risk and environmental exposures lead to autoimmune responses that subsequently evolve into musculoskeletal pain and arthritis. During this at-risk phase, disease prevention may be possible.

**WHAT THIS STUDY ADDS**

- We review current knowledge on how the mucosal surfaces and adaptive immunity influence the development of RA, and how this potentially can be targeted in novel interventional efforts.

**HOW THIS STUDY MIGHT AFFECT RESEARCH, PRACTICE OR POLICY**

- Although we present several recent advances in understanding RA development, considerable knowledge gaps remain. Disease heterogeneity is considerable also before onset, and multinational collaboration could allow more precise subgrouping of study subjects as well as more swift evaluation of novel treatment strategies in clinical trials.

## Introduction

 Even though clinical and radiographic outcomes have improved in recent decades, patients diagnosed with rheumatoid arthritis (RA) today still face the expectancy of a lifelong disease with limited chances of experiencing sustainable drug-free remission.^1^ However, there is tremendous research activity on how and when RA develops, where a deeper understanding of events underlying arthritis onset holds promise for future means to prevent, postpone or at least attenuate the consequences of an RA diagnosis. The processes leading up to a clinical RA diagnosis remain incompletely understood but are believed to comprise a chain of events that take place during several years, many of which may be asymptomatic. The characteristics during this prephase may differ across patient subgroups in keeping with the considerable heterogeneity seen in disease course following diagnosis. For instance, not all individuals seem to experience a prolonged symptomatic prearthritis phase, as shown in a Dutch study comparing early RA patients who had previously presented with clinically suspected arthralgia to those who had not. Here, patient clustering identified an older and more often anticitrullinated protein antibodies (ACPA)-negative subgroup of patients with a subacute onset of symptoms.^2^ Given the rapidly increasing number of studies within the field of pre-RA research, and the substantial impact that successful preventive strategies would have, there is a need to review the current knowledge on potential steps leading to clinical disease and how these mechanisms potentially can be targeted to prevent RA onset.

Individuals without clinical signs of arthritis, but for different reasons are facing an increased risk for RA (eg, by testing positive for RA-related autoantibodies, presenting with small joint arthralgia, or being a first-degree relative (FDR) to a person with RA), are denoted at-risk subjects. This contrasts with the term pre-RA, which is only to be used for cases where clinical RA is known to have occurred.^3^ The risk of progression from at-risk to clinical RA is substantially different depending on the (combination of) factors underlying the at-risk classification, ranging from 0.2% to 57% over a year (table 1), highlighting the significant heterogeneity contained within the term at-risk. Stratifying the risk of progression among at-risk subjects is an important, but challenging, task to achieve personalised management of at-risk patients both in rheumatology and primary care settings. Importantly, risk stratification criteria were recently developed by the American College of Rheumatology and the European Alliance for Rheumatology Associations, which can be used for defining more homogenous risk groups in clinical trials.^4^ It is likely that the characterisation of events that determine the transition from risk phase to overt disease would not only identify potential therapeutic targets but also likely improve individualisation of patient care.

**Table 1:** Approximations of 1-year risk of developing rheumatoid arthritis (RA) in different contexts

Population	Estimated risk	Reference(s)
General (Western) population	0.04%	^153 154^
First-degree relatives to RA patients	0.2%	^155 156^
ACPA-negative with musculoskeletal pain	0.8%	^157^
ACPA-positive in general population	2.9%	^158^
ACPA-and RF-negative clinically suspect arthralgia	8%	^14^
ACPA-positive musculoskeletal pain	15%–42%	15159 161
ACPA-and RF-positive clinically suspect arthralgia	57%	^14^

ACPA, anticitrullinated protein antibodies; RF, rheumatoid factor.

**Figure 1:**
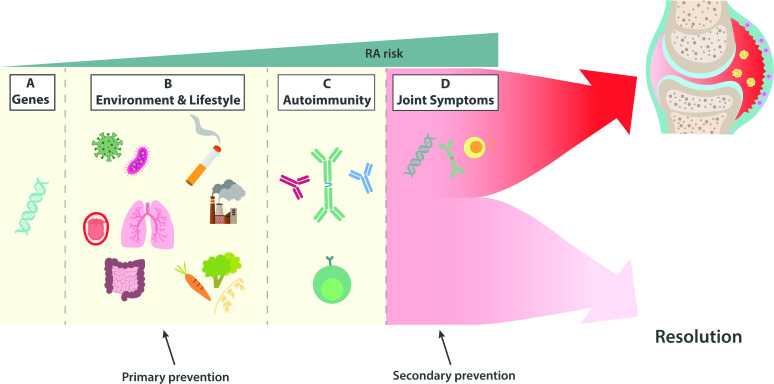
Summary of at-risk phases in the development of rheumatoid arthritis (RA). Risk gene carriage at birth (**A**) is followed by environmental exposures such as mucosal irritants and/or microbes (**B**). Such exposures contribute to autoimmune responses from both the T cell and B cell compartment (**C**). Joint pain subsequently develops by yet unknown mechanisms, without evidence of inflammation within the joint (**D**). In a proportion of subjects, autoimmune responses become directed to the joint during the symptomatic phase and arthritis develops (**D**). A substantial proportion of subjects do not develop arthritis and symptoms resolve (**D**). The breadth of the autoimmune response and the carriage of risk genes in HLA-DRB1 are risk factors for arthritis development, but the exact mechanisms behind transition to the joints remain obscure.

The stages of RA development are summarised in figure 1. In the first phase, individuals lack clinical symptoms and signs of autoimmunity but carry risk genes and possible epigenetic alterations.^5^ In addition, environmental exposures (eg, mucosal irritants or microbes) facilitate a breach of immune tolerance, leading to the occurrence of autoantibodies in the circulation^6 7^ as a result of autoreactive B and T cells having encountered their cognate antigen(s).^8 9^ For most patients, this is followed by years without either symptoms or clinical or histological signs of joint inflammation,^6 7 10^ sometimes referred to as the ‘non-articular’ phase of RA development.^11^ Here, a maturation and broadening of the autoimmune response is believed to take place, illustrated for instance by ACPA which are suggested to increase in levels, expand the number of epitopes recognised and attain a more unfavourable glycosylation pattern.^12^ Eventually, the joints become involved with arthralgia and/or stiffness, which in a subset of subjects develops into clinically overt arthritis. The autoantibody-positive symptomatic period prior to arthritis onset is on average slightly shorter (1–3 years)^13^,^15^ than the asymptomatic autoimmune phase (3–5 years),^6 7^ but both are subject to considerable interindividual variation. Importantly, for each step in the at-risk phase, a significant proportion of individuals do not progress, but the more steps completed, the higher the risk of RA onset (table 1).

Several clinical trials have been conducted in different at-risk populations to test the hypothesis that progression to RA can be halted. Most of these trials have used drugs in current use for manifest disease, that is, drugs designed to dampen ongoing active inflammation. This is not a prominent feature of the at-risk phase, which potentially explains why most trials failed to show preventive effects that last after treatment was stopped. Nevertheless, 12 months of abatacept treatment (ie, inhibition of T cell costimulation) was shown to reduce progression to RA among ACPA-positive symptomatic subjects. However, the effect size was limited 1 year after treatment cessation,^16^ suggesting that the treatment did not substantially alter the long-term course. Instead, novel modes of action appear warranted for the prevention of RA, and for this to take place, a deeper understanding of underlying mechanisms is essential. Aberrations in adaptive immunity are a central feature in the at-risk phase for most pre-RA subjects, and the influence from mucosal surfaces in this context has been increasingly revealed in recent years. With this focus, we set out to overview current knowledge on pre-RA mechanisms and how they potentially can be targeted for preventive purposes.

## Mucosa-related mechanisms in RA development

Although not formally proven, several indications (often collectively termed the ‘mucosal origins hypothesis’) imply that mucosal surfaces play substantial roles in the journey from risk gene carriage to manifest disease.^17^ The bulk of available data addresses mechanisms in the oral cavity, the lungs and the gut, as detailed in separate sections below. In addition, there are some general mucosa-related observations that are not clearly related to a particular mucosal compartment but still merit mentioning. Circulating IgA class ACPA arise prior to symptom onset in a substantial proportion of patients (30%–40%)^18^ and are prognostic for RA onset both before and after symptom onset.^18 19^ IgA responses are often ‘associated by default’ to mucosa even though most of the IgA found in circulation originates from long-lived plasma cells in the bone marrow. Circulating IgA is mainly monomeric and lacks secretory component (SC), that is, the remnant of the poly-immunoglobulin receptor (pIgR) responsible for immunoglobulin transport across epithelial surfaces. In contrast, IgA at mucosal sites is mainly dimeric and complexed with SC, making it highly resistant to degradation.^20^ Interestingly, free SC (ie, soluble pIgR) as well as SC-containing immunoglobulins can be found in the circulation, with increased levels both in symptomatic ACPA-positive pre-RA and at RA diagnosis.^21 22^ Together with the findings that the proportion of circulating IgA+plasmablasts was strikingly increased among seropositive at-risk subjects,^23^ it appears that mucosal antibody responses, and possibly transepithelial immunoglobulin transport, are increased prior to arthritis onset. This upregulation also includes autoantibodies, as SC-containing ACPA arise in the circulation prior to joint symptoms,^24^ predict arthritis onset among symptomatic subjects,^25^ and subsequently appear enriched in RA synovial joint fluid.^22^ It needs to be pointed out, however, that circulating IgA- and SC-ACPA responses mainly occur in a subset of IgG ACPA-positive individuals, and do not clearly precede the IgG response.^18 24^ Therefore, mucosa-associated autoantibody responses do not appear to be a prerequisite for developing a systemic IgG ACPA response, at least not as mirrored in the circulation.

IgA immune complexes (ICs) are present in RA joint fluid and were shown to exceed IgG ICs in their ability to activate neutrophil production of reactive oxygen species r and Neutrophil Extracellular Traps (NETs) ^26 27^, as well as osteoclast bone degradation.^28^ These effector functions, together with their negative prognostic potential in the prearthritis phase, indicate that intervening with IgA-containing or SC-containing IC-mediated pathways may have therapeutic potential. Binding of IgA to the FcαI receptor (FcαIR; CD89) may elicit several different cell responses. In general, binding of polymeric IgA or IgA ICs to FcαIR causes proinflammatory responses, while non-IC monomeric IgA has inhibitory effects.^29^ Therefore, restraining IgA IC binding to Fcα receptors, for instance by anti-CD89 antibodies or saturating CD89 with monomeric IgA, are potential therapeutic options to avoid triggering of arthritis.^30 31^ Also, since IgA glycosylation patterns have proved important for effector functions,^32^ increased IgA sialylation could hypothetically reduce progression to arthritis. Finally, strategies to avoid IgA-promoting events at specific mucosal sites would be of obvious therapeutic potential, as discussed in separate sections below.

### The oral cavity

There has been a longstanding interest in connections between dental health and RA. In later years, the hypothesis of a ‘tooth-joint axis’ has been refined and predominately addresses periodontitis and changes in the oral microbiome. Recent meta-analyses concluded that there are epidemiological connections between RA and periodontitis,^33^ and that non-surgical treatment of periodontitis reduces disease activity in concurrent RA.^34^ It can, however, still be argued that periodontitis is a consequence rather than cause of (or risk factor for) RA. For instance, this could be facilitated by increased systemic expression of proinflammatory cytokines and matrix metalloproteinases (MMP), increased priming of immune cells, reduced ability to control oral pathogens due to immunomodulatory treatment or difficulties with oral hygiene practices due to hand function impairments. Hence, evidence from risk phases of RA is crucial to determine the mechanistic interrelations and identify possible therapeutic approaches. The prevailing hypothesis is that oral bacterial species associated with periodontitis cause a breach of self-tolerance to citrullinated proteins by either molecular mimicry or by their intrinsic capacity to induce citrullination, as shown for *Porphyromonas gingivalis (P.g*.) and *Aggregatibacter actinomycetemcomitans (A.a*).^35^ Serological responses to *P.g*., *A.a* and other periodontitis-associated bacteria have been studied in several at-risk settings. Antibody levels to *P.g*. were found elevated in most,^36^,^38^ but not all studies.^39^ For *A.a*, the picture is mixed both regarding whether antibody levels are increased and if they are prognostic for RA onset.^40 41^ While serological analyses are informative to investigate whether a particular oral bacterium has been encountered sufficiently to mount a systemic immune response, they do not reliably reflect ongoing colonisation or abundance and are certainly not diagnostic for periodontitis. Therefore, studies on the oral microbiome and prevalence of periodontitis in the at-risk phase have been pursued, but with rather disparate findings. In the UK, both *P.g*. and periodontitis were found over-represented among ACPA-positive symptomatic at-risk patients,^42 43^ while a Dutch study on seropositive arthralgia instead found increased abundance of *Prevotella* and *Veillonella* species, without signs of increased periodontitis prevalence.^44^ In a Colombian study on 119 FDRs to RA patients, periodontitis prevalence and severity was increased compared with matched controls, while *P.g*. abundance was not.^45^ Importantly, these studies either lack follow-up data on future RA development or were insufficiently powered for such subanalyses.^46^ On the other hand, by investigating surrogate markers for periodontitis such as self-reported previous tooth extraction^47^ and radiographic marginal jawbone loss,^48^ associations with future RA have emerged, especially in ACPA-positive subjects.

Although existing data from the at-risk phase of RA is somewhat discordant concerning the over-representation of periodontitis and its potential relation to RA progression, the results suggest that the link between RA and periodontitis occurs before arthritis onset. Hence, there is obvious potential in modifying oral microbial imbalances, that is, dysbiosis and/or treating periodontitis to halt progression to RA. *P.g*. remains the prime bacterial suspect in the link between the two diseases but is frequently detected also in the general population,^49^ and thus exposure seems difficult to avoid. There is no vaccine available against *P.g*, but promising candidates have been described.^50^ Protection from *P.g*. colonisation or infection by vaccination could potentially not only offer protection against periodontitis, but also against RA development among at-risk subjects, either by preventing periodontal disease or, regardless of periodontitis, by protecting against autoimmune responses elicited by *P.g*. infection. However, if such vaccination is to prevent autoimmune responses such as ACPA formation, it needs to be given early in the pre-RA phase, where risk for progression is generally low, and where individuals are unlikely to have joint or gum symptoms. Another treatment strategy could instead be to avoid triggering arthritis by periodontitis acting as a ‘second hit’ among ACPA-positive symptomatic subjects (figure 1, section D). Studies on serum antibody levels in different at-risk phases do not imply that bacterial exposure increases as RA approaches,^37 51^ but longitudinal studies on periodontitis and oral microbiome in the at-risk phase are warranted to disentangle optimal strategies for novel treatments. Periodontitis can already today be improved clinically by non-surgical treatment strategies, whereby the periodontal bacterial load is significantly reduced.^52^ Thus, studies evaluating non-surgical periodontitis treatment for the prevention of RA are awaited with great interest. Patient selection in terms of at-risk phase, stage of periodontitis and oral bacterial composition is likely to be of great importance, but studies on ACPA-negative subjects will be difficult due to inherently low progression rates. Efforts to improve dental health in the general population, however, would serve as a potential primary prevention measure (figure 1, section A and B) reaching also seronegative risk individuals.

### The lung

It is well established that RA-associated autoantibodies may develop many years prior to disease onset, and here the lung is of considerable interest given the association between different airway irritants (smoking, silica dust, air pollutants) and seropositive disease^53^,^55^ as well as the increased content of citrullinated proteins in the lungs of smokers.^56^ Hence, smoking cessation (and avoidance) is a lifestyle intervention that rheumatologists generally inform on also to risk subjects. Still, evidence that smoking cessation reduces RA risk is observational^57^ and so far not available specifically from at-risk populations.

When it comes to lung studies in the risk RA phase, Demoruelle *et al* studied 42 at-risk individuals and reported increased airway changes by high-resolution CT as compared with control groups (76 vs 33%).^58^ Two of the at-risk individuals with such lung changes progressed to classifiable RA within 13 months of inclusion, again providing circumstantial evidence of a link between the lung, systemic autoimmunity and transition to RA onset. Studies on induced sputum have also been used as a proxy for the lung. Okamoto *et al* studied samples from 49 at-risk subjects focusing on neutrophils and found increased spontaneous NET formations in the risk subjects versus controls, suggestive of an inflammatory mechanism contributing to break of tolerance and autoimmunity development.^59^

In early RA patients, the relative proportion of IgA as well as IgG ACPA has been reported to be enriched in bronchoalveolar lavage (BAL) fluid,^60^ giving support to local production of ACPA in the lung. Noteworthy here is the (switch) order of the immunoglobulin genes where human IgG+B cells are relatively likely to continue switching into IgA.^61^ So far, there are no studies of ACPA (IgG nor IgA) in BAL fluid from at-risk individuals. However, IgA1 ACPA levels in the circulation have been shown to associate with subsequent RA progression, while IgA2 ACPA did not.^19^ Moreover, such IgA1 ACPA levels were shown to decline in follow-up samples closer to RA onset, suggesting that these autoantibodies may transit to the joint (or other tissues) in conjunction with disease development. Here, more studies in the IgA versus IgG space are warranted.

Cellular lung studies are challenging and invasive, where BAL fluid samples provide a representation of the distal airways. In a recent study of BAL samples from both at-risk and early RA, IgG-switched B cells were the major B cell subset among ACPA+subjects.^62^ Moreover, elevated B cell levels were reported in ACPA+subjects as compared with ACPA−, which together implicate the lung as an active site for adaptive immune responses in subjects with citrulline autoimmunity. Upon sequencing the overall IgG B cell repertoire, the risk subjects had lower levels of somatic hypermutation (SHM) compared with samples from ACPA− early RA, a finding that has been previously reported among circulating B cells comparing ACPA+ and ACPA− patients with established RA.^63^ This emphasises that the general B cell repertoire is not particularly hypermutated in RA, while high SHM is a feature of ACPA+B cells also in the lung.

When generating monoclonal antibodies from the BAL-derived BCR sequences, ACPAs could be identified from both at-risk and early RA.^62^ Interestingly, all ACPAs originated from IgG+B cells and none from IgA+B cells. As for the differentiation state of the citrulline (cit)-reactive B cells, we saw a mix of classical memory B cells and plasmablasts as well as double negative B cells (implicated in so-called extrafollicular B cell responses).^64^ The two monoclonal ACPAs generated from the RA at-risk phase were both derived from memory B cells, that is, not actively secreting antibodies, implicating that their primary role in the lung is antigen-selective uptake via BCR and subsequent antigen-presentation to T cells, perhaps in the inducible Bronchus-Associated Lymphoid Tissue (iBALT)^65^?

To summarise, there are several reports supporting the hypothesis that the lung is involved not only in early RA but also in the risk phase. How common this is among at-risk individuals remains to be established. An intriguing possibility is that the lung could be part of the so-called second hit, that is, not the initial breach of tolerance but the place where the immune response matures before transitioning to the joint. Here, efforts in minimising T cell-B cell crosstalk could be attractive, based on the ability to identify at-risk individuals with lung involvement, as this may not be true for all. To our knowledge, there are no ongoing clinical trials specifically targeting RA-promoting events in the lung during any at-risk phase. One early study showed that short-term treatment with systemic corticosteroids was able to reduce autoantibody levels in symptomatic seropositive subjects,^66^ so local treatment with inhalation steroids could potentially be an attractive and safe option to attenuate autoimmune responses elicited in the lung. From a primary prevention perspective, societal strategies to reduce exposure to airway irritants could bear the potential to decrease RA risk.

### The gut

A gut-joint axis has been suspected for many years, although mainly in the context of spondyloarthritides. Nevertheless, several links with relevance to RA have been around for decades. For instance, Trollmo *et al* showed already in the 90s that oral immunisation yielded antigen-specific antibody-producing cells in the synovium of RA patients.^67^ Although there is still no causal evidence that RA is triggered in the gut, several intriguing findings have recently been made regarding how the microbiome and the epithelial barrier function may influence immune reactions locally in the gut, and the subsequent spreading to joints.

In manifest RA, the gut microbiome has provided consistent findings of imbalances, that is, dysbiosis, when compared with healthy and disease controls.^68^ There are, however, striking disparities concerning which specific bacteria are enriched, and no RA-specific pattern has been confirmed to date. Also, a deviant gut microbiome is not an RA-specific phenomenon, since it is found in several other diseases.^69^ Among specific bacteria, *Prevotella copri* (*P.c*) remains the most prominent among suspects in RA,^70^,^72^ but still fails to show overabundance across all studies,^73^,^75^ and serum antibodies to *P.c* were not significantly elevated in ACPA-positive at-risk patients.^76^ *Prevotella* was found to be increased in faecal samples from at-risk subjects selected on the presence of symptoms and autoantibodies,^77 78^ but not among subjects recruited as FDRs.^79^ On the other hand, *Prevotella* was enriched in a population-based cohort with increased genetic risk for RA.^80^ There are also other species suggested to be involved, as Chriswell *et al* showed that monoclonal autoantibodies from at-risk subjects cross-reacted to the *Lachnospiraceae/Ruminococcaceae genus*, and that such bacteria isolates from faeces elicited CD4+T cell activation to a greater extent in RA patients than among controls.^81^

Intriguingly, studies have shown that HLA genetic variants are important in shaping the gut microbiome in a healthy state,^80 82^ implying that RA genetic risk may partly be mediated via the gut microbiome. If so, modulation of gut dysbiosis would be a potential means to halt progression from the asymptomatic genetic at-risk phase to the asymptomatic autoimmune phase (section C in figure 1). Somewhat discordant to the notion that dysbiosis is a very early event, and genetically determined, Rooney *et al* found that the gut microbiome composition was more dynamic in symptomatic ACPA-positive at-risk subjects who progressed to arthritis as compared with those who did not, and that shifts in the microbiome occurred close to arthritis onset.^78^

**Figure 2:**
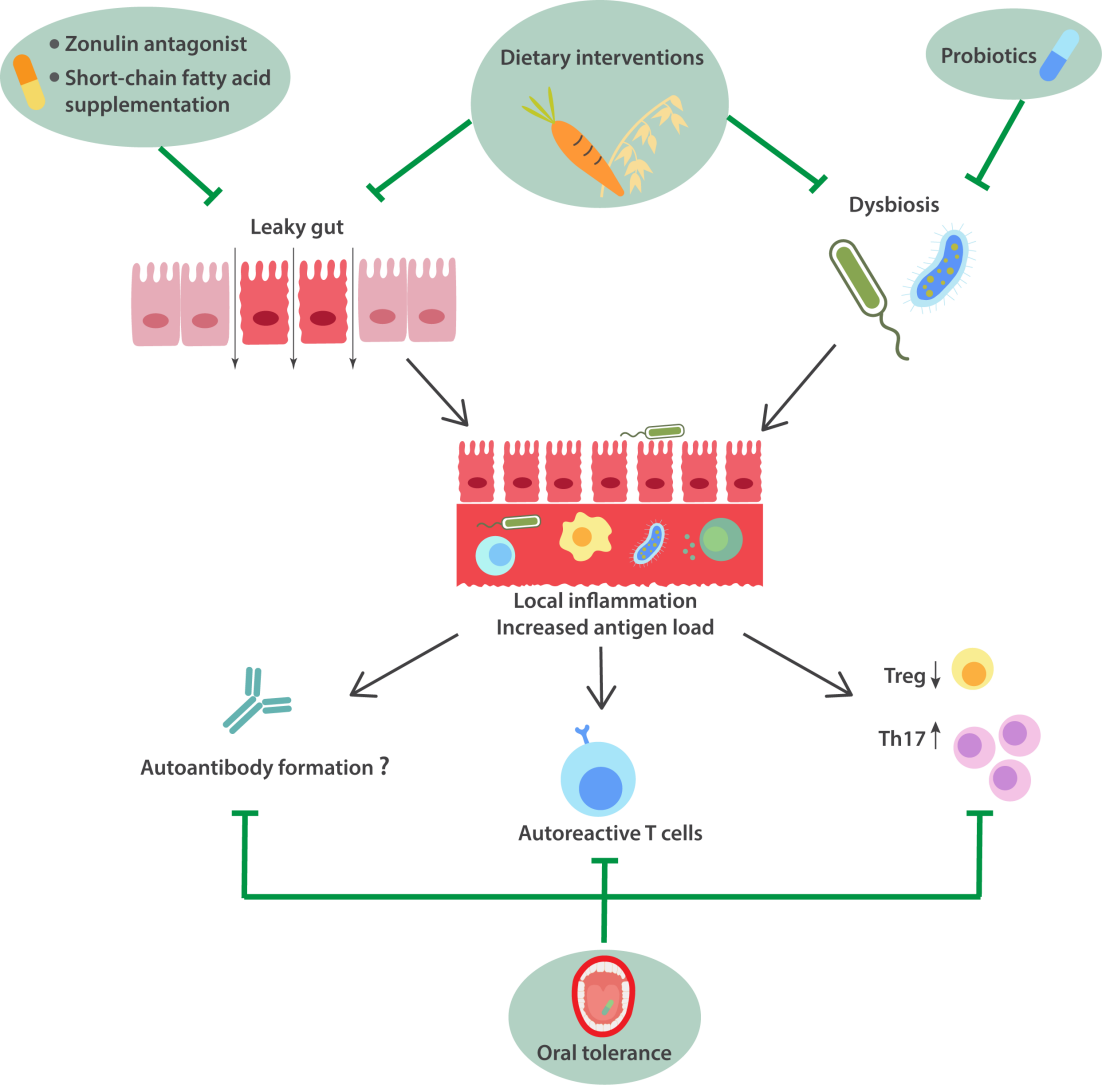
Gut involvement in RA development and potential means to intervene. Increased intestinal permeability and aberrant gut microbiome have been shown prior to RA onset. This allows for increased entrance of antigenic substances from the lumen. Also, translocation of microbes and microbe-associated molecular patterns causes local inflammation and increased risk that self-tolerance is lost and autoreactive T cells are activated. There is downregulation of regulatory T cell responses and an increase in T helper 17 activation. Animal studies showed that autoantibodies may be formed, but human studies have been unable to show local production of RA-related autoantibodies in the gut. Potential means to intervene with gut involvement are in light green.

There are several potential mechanisms by which dysbiosis can contribute to RA, either individually or acting in concert (figure 2). Importantly, dysbiosis may cause disruptions in the host’s intestinal barrier function, causing a ‘leaky gut’.^83^ Normally, the intestinal barrier protects against potentially noxious agents within the lumen by several different mechanisms, and gut immune surveillance is managed without mounting profound systemic immune responses. However, if intestinal barrier functions deteriorate due to dysbiosis, environmental exposures or poor diet, microbes and microbe-associated molecular patterns can translocate into the subepithelial space and elicit local proinflammatory responses. Importantly, this can trigger autoreactive adaptive immune responses when self-antigens are presented in an inflammatory context, causing the occurrence of autoreactive Th17 cells and IgA autoantibody production.^84 85^ In the context of RA and its prephases, there are several indications of barrier function disruptions. For instance, transplantation of faeces from pre-RA subjects, but not from healthy controls (HCs), caused increased intestinal permeability and exacerbation of arthritis in animal models.^75^ Tajik *et al* showed that the expression of proteins important for intestinal barrier function, such as claudin and occludin, was reduced in ileal biopsies from recent-onset RA patients, and that lactulose/mannitol tests confirmed increased intestinal permeability.^83^ In addition, the number of macrophages, T cells and B cells was increased as compared with HCs, providing evidence for subclinical local intestinal inflammation.^83^ In the murine CIA model, they found increased intestinal permeability prior to arthritis onset, and by using mice expressing photoconvertible proteins, they showed that CD4+T cells migrated from small intestines to arthritic joints. Intriguingly, this cell migration and subsequent arthritis could be prevented by restoring gut barrier function.^83^ In another study, hypoxia-inducible factor 2 alpha (HIF2a) was found to be upregulated in ileal epithelium from ACPA-positive at-risk subjects, and in the CIA model, such upregulation was found to mediate barrier dysfunction via increased expression of the pore-forming Claudin 15. Furthermore, deletion of HIF2a inhibited arthritis in the mouse model.^86^

Zonulin is an enterotoxin released from enterocytes in response to microbial or dietary stimuli, which disintegrates barrier functions and increases intestinal permeability. Zonulin-family peptides can be detected in the circulation where levels correlate with intestinal permeability.^87^ Serum levels were found to be increased in two independent cohorts of ACPA-positive symptomatic at-risk patients as compared with healthy as well as ACPA-negative arthralgia patients and were prognostic for arthritis onset in one of the cohorts.^88^ However, there was no correlation between intestinal permeability and ACPA levels, and in contrast to the oral cavity and the lungs, the gut does not appear to be a site of autoantibody production since neither rheumatoid factor (RF) nor ACPA are found in samples from jejunum, ileum or stool.^89 90^

Short-chain fatty acids such as acetate, butyrate and propionate are formed when dietary fibres are digested by intestinal microbes. These compounds have anti-inflammatory and immune-regulating properties,^91^ restore intestinal barrier functions, and mitigate migration of immune cells from the gut to the joints.^83^ Epidemiological studies show that low intake of dietary fibre associates with an increased risk of developing RA,^92 93^ and low serum SCFA levels predict arthritis onset among at-risk patients.^94^ In animal models, high-fibre diet or SCFA supplementation inhibits arthritis,^83 95^ but this has not yet been tested in the at-risk phase in humans. Dietary fibre supplementation in established RA showed that circulating levels of SCFA and regulatory T cells (Tregs) increased, while IgA levels decreased.^96^

Although completely lacking in subjects at risk, there are extensive data on different dietary regimens and supplementations tested in manifest RA (reviewed in Philippou *et al*^97^). Notably, 7–10 days of fasting are associated with rapid and substantial clinical improvements in RA disease activity that are not explained by increases in cortisol.^98^ Unfortunately, the effects do not last irrespective of the diet patients return to,^99 100^ making it an even less feasible treatment option. However, a deeper understanding of the underlying mechanisms could reveal possibilities to address them by other interventions. For now, however, it provides proof of concept that exposures to the gastrointestinal (GI) tract can profoundly influence joint health.

There is another mechanism by which immune reactions could be downregulated by the gut, known as oral tolerance. This process, designed to maintain tolerance to large quantities of harmless food and commensal antigens, involves the induction of Tregs and downregulating cytokines such as TGFβ and IL-10.^101^ Clinical trials have shown significant but moderate therapeutic responses in RA patients given small doses of collagen II orally, while adverse events were practically absent.^102^,^104^ The reduction of circulating type II collagen antibodies was greatest among treatment responders.^105^ Analogous to this, continuous oral treatment with human polyvalent IgG preparations from large plasma pools,that is, similar to preparations used for intravenous immunoglobulin treatment (ie, containing Fc parts of IgG), reduced serum levels of RF in a mouse model, while no changes were seen for unrelated antibodies.^106^ To our knowledge, such oral IgG treatment has not been tested in a human RA setting. Neither has oral tolerance induction towards citrullinated proteins been evaluated in either murine models or humans.

In conclusion, the gut displays altered microbiome and impaired barrier functions prior to RA onset. The specific microbes and timing vis-à-vis the different at-risk phases (figure 1) remain open questions and important tasks for upcoming studies. In murine models, restoration of barrier function by either zonulin antagonists, SCFA supplementation or increased fibre intake alleviates arthritis. Hence, these are potential new strategies in the prephases of RA (figure 2). Likewise, revisiting the concept of oral tolerance in the context of IgG and citrullinated proteins may identify means to control RA-related autoimmunity and prevent arthritis onset.

## Lymphocyte subsets in the at-risk phase

As the at-risk phase often lacks signs of joint inflammation and overt pathology for long time-periods prior to onset of clinically apparent disease, it would be imperative to find immune aberrations that can be followed in peripheral blood, and there have been efforts also in this direction.

### B Cells in the circulation

Kinslow *et al* took the approach of studying plasmablasts (as a proxy for recently activated B cells) in RA risk individuals and HCs and observed elevated frequencies of IgA plasmablasts in seropositive risk subjects. They also sequenced the plasmablast B cell repertoire of five individuals,^23^ and when expressing BCR sequences as monoclonal antibodies, some of the IgA sequences were confirmed to be ACPAs.

In de Vries *et al*, 18 ACPA+at-risk individuals with arthralgia were phenotyped for B cells at time of inclusion and further subanalysed based on RA progression or not (50:50 distribution).^107^ B cells from the risk phase displayed some of the systemic features seen in established RA but to a lesser degree. Signs of a more activated B cell compartment were evident in the naïve subsets who had signs of early activation (aNAV (activated naïve) phenotype), and in the switched memory compartment where both risk progressors and RA displayed an increased proportion of IgG+CD27-B cells as compared with non-progressors and HCs, respectively. For RA, such a difference was also found for IgA+CD27- B cells. Both subsets could be hypothesised to belong to extrafollicular responses since they did not express the classical memory marker CD27.

Altogether, these studies support the notion of ongoing B cell responses in the at-risk phase similar to those observed after disease onset, implicating cell interactions that potentially could be targeted therapeutically. Notably, this was in fact addressed in the recent abatacept trials, as CTLA4-Ig targets costimulation from antigen presenting cells such as B cells.

### T cells in the risk phase

A founding element in the understanding of the risk of developing RA is the strong genetic association to HLA-DRB1 (position 71, 74 and 11), but also single polymorphisms in the HLA-B and HLA-DPb loci. All influencing the peptide binding groove of the respective HLA molecules, hence providing a link to antigen presentation to both CD4+ and CD8+ T cells. This is a refinement of the shared epitope hypothesis, where the shared epitope alleles remain high-risk alleles for ACPA+disease.^108^ Notably, the impact of HLA appears to be important in the progression to disease among ACPA+individuals, rather than for the development of ACPA,^109 110^ underscoring the importance of T cells in this transition.

Whereas T cells are likely to have an important part in the adaptive immune response in seropositive as well as seronegative RA, the mechanisms and triggering events might differ in these groups, exemplified by the limited genetic association to HLA in seronegative disease^111^ and that development of RF is less T cell-dependent than ACPA in seropositive disease.^112^ This influences which conclusions can be drawn from T cell studies in the risk-RA phase, as a mix of either ACPA and/or RF positive individuals has been studied. Notably, disease progression is not always known, and the risk group is thus likely to include individuals who will not develop disease.

#### T cells in the circulation

Ponchel *et al* addressed the role of T cells in the risk phase in a series of studies on symptomatic ACPA+individuals. Here, progressors showed lower frequencies of circulating naïve CD4+T cells, CD8+T cells and regulatory T cells in comparison to non-progressors, whereas a subset denoted ‘inflammation related cells’ (IRC) and regulatory B cells were increased and associated with rapid progression to arthritis onset. The IRC cell subset has not commonly been analysed in other RA settings, but constitutes CD4+T cells with a naïve phenotype expressing CD45RA and CD28, although lacking CD62L expression, which is a feature of memory cells.^113^,^115^

Recently, the T peripheral helper cells have gained much interest in RA, as possible cells to interact with B cells in extrafollicular responses, either in conjunction with germinal centre reactions in secondary lymphoid organs or in the inflamed joints.^116 117^ Circulating T peripheral helper (HLA-DR+Tph) memory cells have been shown to be increased in both ACPA+at risk individuals, pre-RA individuals and RA patients in comparison to HCs. Although tested in a very small cohort, these cells were also significantly increased in progressors (n=4) versus non-progressors (n=13).^118^ However, no difference was seen regarding Tph cells (CD4+CXCR5^−^PD1^hi^) in progressors (n=10) vs non-progressors (n=10) in a study on HLA-DRB1*04:01 ACPA+risk individuals.^119^ This difference may relate to variation in the inclusion criteria of the cohorts, and that slightly different Tph cell subsets were assessed.

Immunological studies in the risk phase hence address both pathophysiology and potential biomarkers. There are promising results in the quest for the latter, to determine disease progression, although further studies are needed for confirmation.

#### Autoreactive CD4+ T cells can be detected in the circulation prior to disease onset

The occurrence and phenotype of circulating antigen-specific T cells recognising citrullinated antigens have been studied in three different settings: in ACPA+at-risk individuals (progression rate unknown),^8^ ACPA+at-risk individuals (progressors and matched non-progressors)^119^ and FDRs to RA subjects.^120^ HLA-class II tetramers loaded with different citrullinated antigens were used to capture the autoreactive CD4+T cells in individuals with a specific HLA type (eg, HLA-DRB1*04:01). Although rare, such autoreactive T cells can be detected in the circulation before disease onset. James *et al* showed that the total number of citrulline-specific T cells, and cells specifically recognising citrullinated cartilage intermediate layer protein (cit-CILP) were more frequent in at-risk individuals compared with controls, and that the CILP-specific cells were of a Th17 phenotype.^8^ Comparable frequencies of cit-specific T cells were detected by Turcinov *et al,* but surprisingly they were decreased among progressors compared with non-progressors. Possibly, this finding indicates ongoing homing to lymphoid or joint tissue.^119^ In this study, citrullinated tenascin C was the most frequent autoantigen among the progressors,^119^ in line with previous results from established RA.^121^ In contrast, the FDRs of indigenous north American RA patients had similar frequencies in antigen-specific T cells recognising both native and citrullinated vimentin. The diverging results might be related to differences in HLA genetics and background RA risk between cohorts.^120^ Additionally, the fact that only partly overlapping fine specificities were analysed in the different studies precludes the possibility of making an overall conclusion regarding which T cell autoreactivity is dominating in the at-risk phase and in individuals progressing to RA.

Investigating the T cell receptor repertoire is a different approach to understanding possible disease drivers, where data in the risk phase is still limited. A trend towards higher frequency of highly expanded T cell clones in symptomatic FDRs was seen in one study, driven by a subset of individuals with undifferentiated arthritis (UA).^122^ Notably, assessment was done on the TCR-beta chain from genomic DNA in whole blood, without the possibility to determine CD4+/CD8+status.

To summarise, previously activated autoreactive CD4+T cells recognising citrullinated antigen:HLA complexes, linking citrulline autoimmunity to genetic risk, can be detected already in the risk phase. The predictive power of assessing such cells in circulation needs to be established by further studies, possibly including different sites (eg, lymph nodes, LNs).

#### T cells in the lymph node: a route to the joint?

Activation of T cells, as well as T and B cell germinal centre reactions, occurs in LNs. A series of studies on LN biopsies have been performed, comparing symptomatic seropositive at-risk individuals to early RA patients and HCs. None of the risk-RA individuals progressed to RA during the follow-up period.^123^,^126^ Anang *et al* showed that while the frequencies of CD4+T cells and CD4+CXCR5+ T-follicular helper cells (Tfh) were higher in RA in comparison to HCs, the at-risk group ended up in-between. In both at-risk and RA, frequencies of CXCR5+CD4+ T cells correlated with CD19+B cells. Additionally, the CD8+CXCR5+ T cells were increased in both at-risk and RA.^123^ Earlier studies did not show any difference in CD4+^124 125^ or CD8+T cells in neither at-risk nor arthritis compared with healthy, but increased frequency of activated CD8+CD69+ cells.^124^ Ramwadhdoebe *et al* found a trend in risk individuals and significantly increased CXCR3+CCR6-CCR4- cells (Th1 profile) in the LN of RA patients compared with HC, whereas there was no difference in IFNγ secretion from CD4+cells on PMA/inomycin stimulation. Instead, decreased IL-4 and IL-10 production was seen in CD4+LN cells from risk individuals in the same setting. Decreased IL-10 in the risk group in combination with increased IL-17 secretion from CD4+T cells in RA patients on in vitro stimulation of PBMC, was together with the LN findings interpreted as a shift in the immunoregulatory balance already in the risk phase.^125^ Cit-specific CD4+T cells were also detected in all LN samples from at-risk individuals (n=5), with cit-vimentin specific cells in 4 out of 5 individuals.^126^ Notably, cit-vimentin/cit-fibrinogen was rarely detected in the circulation of risk-RA individuals in the aforementioned studies by James *et al* and Turcinov *et al*, which might further strengthen the hypothesis of ongoing homing of cells to lymphoid organs.^8 119^ In summary, there are indications of T cell alterations towards B cell interaction and proinflammatory features of both CD4+ and CD8+ T cells in the LNs of at-risk individuals. While conclusions are hampered by the lack of known disease progression in available reports, the LN compartment remains highly interesting as an immunological relay station and a possible site of autoreactive cell activation and interaction.

#### When do the T cells enter the joint?

The risk phase often comprises a period of autoimmunity without manifest arthritis (figure 1), until the point when immune cells do infiltrate the synovium and inflammation becomes evident. By studying a group of at-risk individuals (seropositive with/without symptoms of whom 27% progressed to arthritis), de Hair *et al* showed that the presence of CD3+T cells in synovial biopsies from knee joints (in the absence of overt inflammation) combined with ACPA-positivity was significantly associated with progression to arthritis. Additionally, CD8+T cells in the synovium were associated with having ≥1 ACPA-specificity, although with different specificities than the most frequently found in arthritis progressors.^10^

Traditionally, CD4+T cells have been the T cell in focus in RA pathology, but the role of CD8+T cells is gaining more interest. Refined genome-wide analyses have shown that HLA-class I alleles are also associated with risk of disease,^108^ implicating a role for CD8+T cells in this process. CD8+T cells are indeed found to constitute clonal expansions in the synovium of early RA patients,^127^ but whether this is primarily related to, for example, citrulline reactivity of CD8+T cells,^128^ granzyme K+CD8+ cells,^129^ a bystander recruitment to the inflammatory milieu or the link between a viral ‘second hit’ remains to be elucidated.

**Figure 3:**
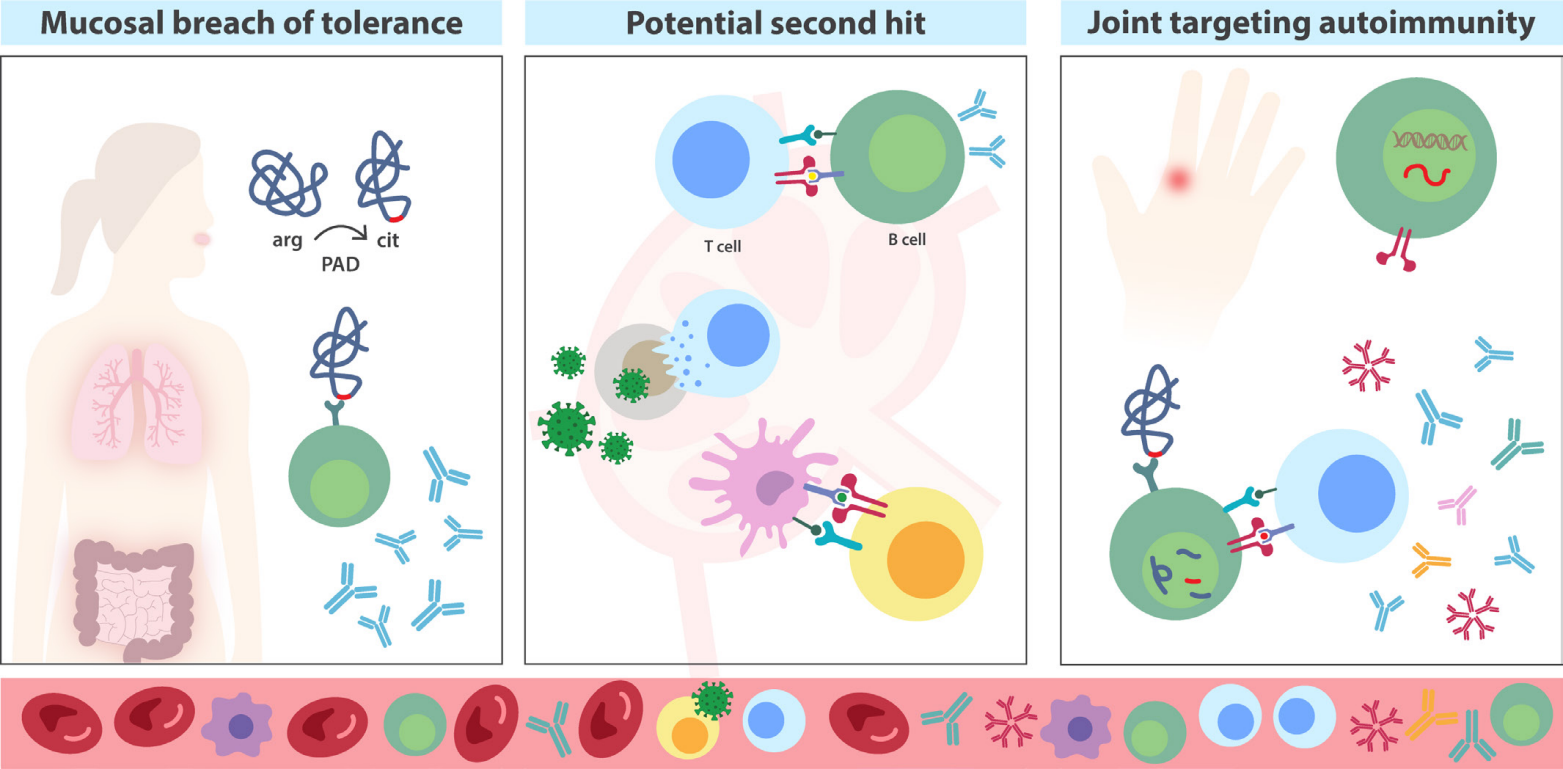
An autoreactive response in three acts: (1) Post-translational modification of arginine (arg) residues via the peptidylarginine deiminase (PAD) enzyme results in the formation of citrulline and possible conformational changes of the protein. Mucosal surfaces constitute a large area for interaction with environmental triggers and citrullination of proteins, providing a site for eliciting an anti-citrullinated protein antibody response. (2) It is unknown what constitutes a ‘second hit’, but even a physiological response (CD8+ and/or CD4+T cells) to, for example, a microbe may provide either an inflammatory milieu or serve as molecular mimicry that promotes an autoimmune response in the at-risk phase. (3) Genetic risk appears to be important also in the transition from systemic autoimmunity to disease, where autoreactive B cells can present and activate autoreactive T cells via HLA, acting as antigen presenting cells, and such T cells can in turn provide the necessary signals for B cell maturation. These events may potentially evolve into the joint targeting of autoimmunity and arthritis onset. The gradual, relative enrichment of immune components observed in the circulation is depicted 'inside the blood stream'

To conclude, there are emerging data on the role of the adaptive immune system in the risk phase of RA development. Trying to put the different events into a trajectory leads us to propose an initial immune dysregulation and systemic autoimmunity in mucosal surfaces, a second hit involving both CD8+ and CD4+ T cells, involving HLA-specific interactions of autoreactive cells resulting in joint targeting responses (figure 3).

### Immune intervention focusing on adaptive immunity

With an increased understanding of antigen-specific immune responses and immune aberrations in the risk-RA phase follows the possibility of interfering with the disease-initiating or driving processes. So far, several studies have ‘repurposed’ RA-approved drugs for the prevention of RA,^130^ but so far no interventions have been specifically developed for the at-risk phase.

Importantly, there is also the recent success of using interventions from oncohaematology based on deep B cell depletion for treating severe refractory rheumatology patients, including RA.^131^ Such immune resetting is too aggressive for the risk RA phase but illustrates the central role for adaptive immunity in RA.

Antigen-specific therapy and the potential for tolerance induction, meaning immune re-education without broad immune suppression, is a very attractive idea provided sufficient knowledge on the disease driving antigens. Such interventions are already being tested in several other autoimmune disease settings such as coeliac disease, multiple sclerosis and type 1 diabetes.^132^ In ACPA+RA, the candidate antigens are still many, and larger studies are needed. Moreover, the site of local breach of tolerance, be that lung, gum, GI or elsewhere, needs to be better understood. But given that these obstacles would be overtaken, the notion of selective silencing only of the RA-initiating adaptive immune responses during the risk phase would be very appealing. This could be achieved, for example, by antigen delivery in a tolerogenic fashion, by so-called tolerogenic dendritic cells, nanoparticles, antigen-specific CAR-Tregs or other means.

## Seronegative disease in the prearthritic phase

Current concepts on RA development are to an overwhelming extent based on data from individuals who test positive for ACPA and/or RF. Also, subjects included in observational at-risk studies as seronegative individuals may later develop autoantibodies, and information on autoantibody status during follow-up is frequently missing. This introduces the risk that a subset of seronegative at-risk subjects in fact represent seropositive disease but are included prior to the systemic appearance of autoantibodies as tested by current assays and cut-off limits. Finally, there is a longstanding debate about whether seronegative disease is, in fact, a true disease entity. For instance, the discovery of additional ACPA reactivities, antibodies towards other post-translationally modified proteins, and other emerging autoantibodies have reduced the proportion of ‘true seronegative’ disease.^133^ Also, what initially may be considered seronegative RA may after longer follow-up end up being more appropriately classified as spondyloarthritis or other rheumatic diseases.^134 135^ From a genetics point of view, there is clear evidence of seronegative RA being distinct from seropositive disease,^111 136^ while more controversy remains concerning differences in heritability between the two subsets.^137 138^ Nevertheless, a genetic basis for seronegative disease seems to exist, and thereby also a seronegative at-risk population (figure 1, section A).

From an immunological perspective, the prearthritic phase of seronegative disease is poorly characterised. Kokkonen *et al* studied circulating cytokines and chemokines in asymptomatic pre-RA and found elevations of multiple proinflammatory markers. Although they were in general substantially less upregulated in the seronegative subset, IL-8 and IL-13 elevations in plasma preceded seronegative RA development.^139^ This could not be confirmed, however, by whole blood RNA analyses in a symptomatic, predominately seronegative, at-risk cohort from the Netherlands.^140^ Instead, they found that IL-7 receptor (IL-7R) was decreased among those who later progressed, independent of serological status. This was in line with findings that a downregulated IL-7 system was predictive of seronegative RA development in an UA setting.^141^ In a follow-up study of the Dutch at-risk cohort, it was shown that IL-7 markers did not change over time until arthritis onset.^142^ Another marker with relevance to seronegative disease is MMP-3, which in serum predicted RA development among seronegative UA patients^143^ and was found specifically upregulated in synovial immune cells in seronegative RA when compared with seropositive disease.^144^ However, although levels were higher in synovial fluid than in serum, Weitoft *et al* did not find differences according to autoantibody status.^145^

Khidir *et al* prospectively compared clinical and imaging trajectories according to serological status in subjects with clinically suspect arthralgia, revealing that seronegative individuals had more symptoms than seropositive subjects but less subclinical inflammation on MRI.^146^ Possible immunological explanations underlying these differences remain largely unknown, but are important questions for future work. In an in-depth characterisation of inflamed synovial tissue from manifest RA, a myeloid pattern was more common among seronegative patients, again suggesting that different mechanisms are at play in seronegative disease.^147^ Finally, a recent multimodal phenotyping of circulating immune cells showed expansion of CD15+classical monocytes specifically in ACPA-negative FDRs, but the predictive value was not addressed.^148^ In conclusion, there is scarce but still increasing knowledge concerning seronegative prearthritic phases. Prospective studies are complicated by low progression rates.

## Discussion

This review outlines several influential mechanisms in RA development, illustrating how genetic risk and mucosa-related breach of tolerance can evolve into joint symptoms and eventually arthritis. Although research efforts on these events have been greatly intensified in recent years, the current body of knowledge remains a patchwork of observations in various at-risk phases and different subsets of at-risk individuals. As evident in this review, results diverge across different at-risk phases and populations, implying that there are many different pathways leading to an RA diagnosis and that searches for one single explanation are likely to be unsuccessful. Personalised medicine seems to be advocated also in the risk phase.

From an interventional perspective, addressing the autoimmune symptomatic phase is the most appealing, as progression risks are the highest and consequently the number needed to treat to prevent one RA patient is the lowest. Also, patients in this phase are for apparent reasons more likely to seek healthcare and, therefore, potentially more motivated to actively participate in any intervention.^149^ It is thus imperative to understand more about the triggers of arthritis onset in high-risk populations such as ACPA-positive individuals with arthralgia to tailor new interventional strategies. Intriguingly, the factors associated with increased probability of progression, for example, levels of circulating autoantibodies and cytokines, do not increase further as arthritis approaches, at least not when studied prospectively.^25 140^ This, together with the fact that up to 50% remain arthritis-free 5 years after presenting with ACPA and musculoskeletal pain,^15^ clearly implies that distinct additional ‘hits’ are required during the autoantibody-positive symptomatic phase to take the disease to the joint. Here, it may be useful to consider local factors in the target organ, that is, the joint, and to revisit the old but still unanswered question why some joints are more prone to be affected than others. Although inflamed joints appear histologically similar within the same RA patient,^150^ there can still be site-specific differences prior to arthritis onset.^151^ Speculatively, non-immunological events in the synovial compartment or cartilage may allow for a preformed systemic autoimmunity to turn to the joints and cause arthritis. Such local triggers could, for instance, be environmentally induced epigenetic alterations or post-translational modifications of proteins that expose novel antigens that can be targeted by already existing autoantibodies.

As our understanding of the autoreactive B and T cell repertoires begins to take shape also in the at-risk phase, another potential approach apart from antigen-specific tolerance could be selective targeting of the autoimmune cells based on the receptor gene usages they deploy. But the repertoires are still only beginning to be delineated.

Interventions prior to the advent of autoimmunity and symptoms, that is, primary prevention, come with several obstacles. First, it will engage large populations, and hence cost-effectiveness will be of major importance. Second, facing a relatively low risk increase of the disease in question may reduce the individual’s motivation to engage in a preventive effort. Third, potential side effects of the intervention need to be carefully considered since many individuals will be addressed who would never have developed RA. Ideally, the interventions in this phase should have additional health benefits in addition to lowered RA risk, for example, reduced risk of cardiovascular and pulmonary disease via smoking cessation, improved dental health by better oral hygiene practices or lowered risks of obesity, cardiovascular disease and malignancy by aligning with dietary recommendations. These circumstances are likely also applicable to individuals with higher risk for RA, such as FDRs or subjects with seropositive arthralgia. Here, surveys and interviews have highlighted that the willingness to participate in preventive efforts sometimes is hampered by concerns for side effects, low efficacy of the intervention or low anticipated RA risk.^152^ In general, lifestyle interventions appear preferred over pharmaceutical substances.

We conclude that mechanisms in play prior to RA onset are being increasingly elucidated, which hopefully will result in novel preventive strategies that can be subjected to clinical trials. This is good news since trials on existing DMARDs in the at-risk phase have not proven substantial preventive potential, and their use in the at-risk phase has not been directly compared with prompt initiation at the time of arthritis onset. Interventions that bring additional health benefits, such as increased fibre intake or treatment of periodontitis, are particularly attractive when treating risk subjects. In high-risk subjects, however, more advanced therapies such as tolerance induction can be advocated. Given the immense activity in the research field, the coming years are likely to improve our map of the pre-RA landscape, and we find particular priority in understanding the step closest to arthritis onset, that is, what takes the disease to the joints. With the growing number worldwide of cohorts with longitudinal follow-up of at-risk subjects, international cross-disciplinary collaborations could not only increase the ability to study specific at-risk phases and defined subsets of individuals with sufficient statistical power, but could also promote more swift evaluation of novel preventive interventions in upcoming clinical trials. Hopefully, these combined efforts will allow future RA treatment guidelines to be more about the pre-RA phase than manifest disease.
